# Subtle Differences in Cognition in 70-Year-Olds with Elevated Cerebrospinal Fluid Neurofilament Light and Neurogranin: A H70 Cross-Sectional Study

**DOI:** 10.3233/JAD-220452

**Published:** 2023-01-03

**Authors:** Maya Arvidsson Rådestig, Johan Skoog, Henrik Zetterberg, Tobias Skillbäck, Anna Zettergren, Therese Rydberg Sterner, Madeleine Mellqvist Fässberg, Simona Sacuiu, Margda Waern, Hanna Wetterberg, Kaj Blennow, Ingmar Skoog, Silke Kern

**Affiliations:** a Center for Ageing and Health (AgeCap), University of Gothenburg, Mölndal, Sweden; b Department of Psychology, University of Gothenburg, Gothenburg, Sweden; c Department of Psychiatry and Neurochemistry, Institute of Neuroscience and Physiology, The Sahlgrenska Academy, University of Gothenburg, Mölndal, Sweden; d Clinical Neurochemistry Laboratory, Sahlgrenska University Hospital, Mölndal, Sweden; e UCL Institute of Neurology, Queen Square, London, UK; f The UK Dementia Research Institute, UCL, London, UK; g Hong Kong Center for Neurodegenerative Diseases, Clear Water Bay, Hong Kong, China; h Psychiatry/Cognition and Old Age Psychiatry Clinic, Sahlgrenska University Hospital, Region Västra Götaland, Gothenburg, Sweden; i Memory Disorders Clinic, Theme Inflammation and Aging, Karolinska University Hospital, Region Stockholm, Stockholm, Sweden; j Department of Neurobiology, Care Sciences and Society (NVS), Clinical Geriatric, Karolinska Institute, Stockholm, Sweden; k Psychosis Clinic, Sahlgrenska University Hospital, Region Västra Götaland, Gothenburg, Sweden

**Keywords:** Biomarkers, cerebrospinal fluid, dementia, neurofilament protein, neurogranin

## Abstract

**Background:**

Most research on cerebrospinal fluid (CSF) neurofilament light protein (NfL) as a marker for neurodegeneration and neurogranin (Ng) for synaptic dysfunction has largely focused on clinical cohorts rather than population-based samples.

**Objective:**

We hypothesized that increased CSF levels of NfL and Ng are associated with subtle cognitive deficits in cognitively unimpaired (CU) older adults.

**Methods:**

The sample was derived from the Gothenburg H70 Birth Cohort Studies and comprised 258 CU 70-year-olds, with a Clinical Dementia Rating score of zero. All participants underwent extensive cognitive testing. CSF levels of NfL and Ng, as well as amyloid β_1 - 42_, total tau, and phosphorylated tau, were measured.

**Results:**

Participants with high CSF NfL performed worse in one memory-based test (Immediate recall, *p* = 0.013) and a language test (FAS, *p* = 0.016). Individuals with high CSF Ng performed worse on the memory-based test Supra Span (*p* = 0.035). When stratified according to CSF tau and Aβ_42_ concentrations, participants with high NfL and increased tau performed worse on a memory test than participants normal tau concentrations (Delayed recall, *p* = 0.003). In participants with high NfL, those with pathologic Aβ_42_ concentrations performed worse on the Delayed recall memory (*p* = 0.044). In the high Ng group, participants with pathological Aβ_42_ concentrations had lower MMSE scores (*p* = 0.027). However, in regression analysis we found no linear correlations between CSF NfL or CSF Ng in relation to cognitive tests when controlled for important co-variates.

**Conclusion:**

Markers of neurodegeneration and synaptic pathology might be associated with subtle signs of cognitive decline in a population-based sample of 70-year-olds.

## INTRODUCTION

Preclinical Alzheimer’s disease (AD) is a common condition in older adults with normal cognition [1]. Preclinical AD is reflected in altered cerebrospinal fluid (CSF) AD biomarkers: amyloid-β (Aβ_42_), total-tau (t-tau), and phosphorylated tau (p-tau). We have previously investigated the performance on cognitive tests in cognitively normal older adults with amyloid and tau pathology (and noted only slight differences between cognitively unimpaired participants with preclinical AD pathology and those without [2]). In this study we sought to look into the performance of the same test battery in those with high concentrations of the biomarkers neurofilament light (NfL) and neurogranin (Ng). In previous research, it has been shown that cognitively normal individuals with underlying amyloid pathology are at greater risk to convert to mild cognitive impairment (MCI) or dementia [3]. As clinical trials for AD include cognitively unimpaired individuals with preclinical AD, it is important to understand if there are subtle cognitive differences present even in this very early disease phase and the relationship between cognition and the newer AD biomarkers NfL and Ng. These biomarkers reflect early pathological changes and have been shown to predict cognitive decline in cognitively unimpaired older adults [4, 5].

NfL is an axonal structural protein, predominantly expressed in large caliber myelinated axons [6, 7]. NfL is released into CSF upon axonal damage and seems to be an unspecific marker as it is increased in AD and MCI [6], but also in other neurodegenerative conditions [8–10], and is associated with increased risk of progression to AD and worsening cognition [11]. CSF NfL was shown in one study to predict hippocampal atrophy rate in cognitively normal older adults, independently of other AD biomarkers [12], indicating that it could possibly play a role in affecting memory functions in the pre-symptomatic phase. However, it is still unclear if NfL correlates with subtle changes in cognition in the preclinical phase.

Ng is a post-synaptic protein, and its CSF levels reflect the degree of synaptic dysfunction in AD [13]. It is expressed in dendritic spines [14] and thought to be involved in memory functions [14, 15]. Ng is highly increased in the regions affected in AD: the cerebral cortex, hippocampus, and amygdala [14]. Increased CSF Ng seems to be specific for AD [16, 17]. Some studies report that Ng is increased in early AD [4, 14, 18], which is consistent with autopsy and biopsy studies showing a considerable synapse loss early in the disease [19–21]. Although much uncertainty still exists about the relationship between synapse loss and neurodegeneration in AD, synaptic loss has been shown to correlate with cognitive decline (Mini-Mental State Examination (MMSE), Delayed recall) and may be the most reliable correlate of dementia [20, 22]. However, it is still unclear if Ng correlates with early changes in cognition.

### Aims

Studies on NfL and Ng in cognitively normal older adults are sparse [4, 5]; to date, research has focused on clinical samples rather than population-based studies. We therefore aimed to investigate the hypothesis that high concentrations of NfL and Ng in CSF are associated with subtle cognitive deficits in cognitively unimpaired 70-year-olds. A secondary aim was to examine if such subtle cognitive differences were present in people with preclinical AD (pathologic CSF Aβ_42_ and/or tau concentrations) and high CSF NfL and/or Ng.

## METHODS

### Participants

The sample was part of the Gothenburg H70 Birth Cohort studies (the H70 studies) and was systematically derived from the Swedish Tax Agency’s population register. Seventy-year-old residents in Gothenburg born in 1944 on specific birth dates (ending with 0, 2, 5, or 8) were eligible for the study and invited to participate (*n* = 1,839) [23]. Of the 1,203 participants examined, 430 consented to a lumbar puncture (LP) (response rate 35.7%) but only 322 underwent LP due to contraindications in 108 (anticoagulant therapy, immune-modulating therapy, or cancer therapy). We based our analyses on a selection of people with a global Clinical Dementia Rating (CDR) score of 0 [24] (*n* = 259) from these 322 individuals. After excluding six individuals with dementia, there were 313 individuals with NfL data and 315 individuals with Ng data. In the resulting CDR0 sample there were 256 persons with NfL and 258 with Ng data.

### General examination

The examinations were conducted at the Psychiatry, Cognition and Old Age Psychiatry Outpatient Clinic at Sahlgrenska University Hospital in Gothenburg, Sweden. They included psychiatric and physical examinations, CSF sampling, blood-sampling for genetic analyses, examinations of social factors, diet, body composition, functional ability, MRI imaging, close informant interviews, and have been described in detail elsewhere [1, 23].

### LP and CSF collection

LP was conducted by a neurologist or psychiatrist. Prior to LP, each participant underwent CT and/or MRI to detect contraindications [23], as described elsewhere [1]. CSF was collected by LP, conducted in the morning, from the L3/L4 or L4/L5 interspace, and transported immediately to the laboratory where it was centrifuged, aliquoted in polypropylene tubes and stored at –80°Celsius. CSF total tau and tau phosphorylated at threonine 181 (p-tau) levels were measured using a sandwich enzyme-linked immunosorbent assay (ELISA) (INNOTEST^®^ htau Ag and PHOSPHO_TAU (181P), Fujirebio (formerly Innogenetics [25, 26].) CSF Aβ_42_ was measured using a sandwich ELISA (INNOTEST^®^ β-amyloid_1 - 42_), specifically constructed to measure the 1–42 isoform of Aβ [27]. The following CSF cut-offs were used to define AD biomarker pathology [28, 29]: CSF Aβ_42_ concentrations ≤530 pg/mL, p-tau concentrations of ≥80 pg/mL, and CSF t-tau concentrations ≥350 pg/mL [1, 30].

CSF levels of t-tau, p-tau, and Aβ_42_ were analyzed as part of clinical routine diagnostics, using established procedures for quality control [31]. CSF aliquots were stored at –80°C pending analysis of NfL and Ng, which were analyzed using the same batch of reagents. CSF NfL [32] and neurogranin [14] were analyzed using in-house ELISA methods developed at the Mölndal Clinical Neurochemistry Laboratory by board-certified laboratory technicians blinded to the clinical data. This procedure has been described in more detail previously [1, 23].

### Cognitive examination

Examinations were performed by experienced research nurses, medical doctors, or a psychologist, and included ratings of psychiatric symptoms and signs, tests of mental functioning, including assessments of episodic memory (short-term, long-term), aphasia, apraxia, agnosia, executive functioning, and personality changes [1, 23].

Additional cognitive assessments were performed by a psychologist, research nurse, medical doctors, or trained research staff, using a neuropsychological test battery, including the following cognitive tests: 1) memory (Immediate and Delayed recall (12 object memory tests), Word memory (10 word memory list), Supra span (10 word memory list (BUSII) [33]), Thurstone’s picture memory test [34]; 2) language (semantic fluency animals, phonetic fluency controlled oral word association FAS); 3) executive function (Figure logic (SRB2), Digit span backwards); 4) visuospatial (Block design, (SRB3)); and 5) mental speed (Psif), all of which have been described elsewhere [23, 35, 36]. This battery covers the cognitive domains memory, language, visuospatial ability, executive function, and mental speed [2].

Global cognitive status was assessed by a Swedish version of the MMSE [37] and the assignment of a CDR score [24, 38], by research nurses or by a geriatric psychiatrist/neurologist [2].

Dementia was diagnosed according to the DSM-III-R [39] criteria as previously used in the Gothenburg H70 Birth Cohort studies, and major depression was diagnosed according to DSM-5 [40]. Education (defined as years of education) and history of stroke and transient ischemic attack (TIA) was acquired from self-reports and close informant interviews. Close informants and participants were also asked about family history of dementia, depression, and stroke [23].

### APOE *ɛ*4 genotyping

The single nucleotide polymorphisms (SNPs) rs7412 and rs429358 in *APOE* (gene map locus 19q13.2) were genotyped, using KASPar^®^ PCR SNP genotyping system (LGC Genomics, Hoddesdon, Herts, UK). Genotype-data for these two SNPs were used to define *ɛ*2, *ɛ*3, and *ɛ*4 alleles [1]. Data on *APOE* genotype was lacking for five individuals.

### Ethical considerations

All participants and/or their key informants provided written informed consent. The H70 study was approved by the Regional Ethical Review Board in Gothenburg and conducted in accordance with the declaration of Helsinki [23].

### Statistical analyses

Since the NfL variable was not normally distributed it was log transformed (log-10) prior to these analyses. This was also done with the Ng data, which was slightly skewed. Since there is no established pathological cutoff for CSF- NfL levels in unimpaired populations of older individuals and cut-offs are generally derived from patients with dementia, participants were divided into groups based on the median of CSF-NfL and CSF-Ng. Differences in sample characteristics were analyzed using Student’s T-tests for continuous variables and Fischer’s exact test for categorical variables. T-tests were performed to compare the results on cognitive tests between individuals with NfL above the median and NfL below the median. The same procedure was applied to analyze cognitive test results in relation to Ng. In addition, we compared neuropsychological test scores for participants with and without preclinical AD, using T-tests, in subsamples with high and low NfL levels. We used NfL and Ng as binary variables. Further, the cohort was divided into tertiles according to CSF-Ng and CSF-NfL concentrations, and we performed T-tests comparing cognitive test performance between the tertiles, with the bottom tertile as reference group. We also divided the participants according to amyloid and tau concentrations, where pathologic tau concentrations was defined as a combination of T-tau and P-tau in order to avoid small groups.

To adjust for potential covariates, linear regressions were performed with cognitive test performance as dependent variable, and NfL, age, education, sex, and *APOE* *ɛ*4 status as independent variables. The same procedure was repeated for the Ng variable.

A nonparametric sensitivity test (Mann-Whitney U-test) was also conducted comparing cognitive performance in individuals with NfL above the median with individuals with NfL below the median, and Ng above versus below the median. We also performed Hochberg correction for multiple testing and *post-hoc* power analyses.

A two-tailed level of significance was used for all analyses (*p* < 0.05). Statistical analyses were performed in IBM SPSS Statistics for Windows (version. 25.0, Armonk, NY: IBM Corp), and Stata, version 14.0, StataCorp, Texas, USA. Normal distribution was assessed graphically and with Shapiro-Wilk’s test. The tau pathology variable contains some individuals with amyloid pathology and viceversa.

### Power analysis

A *post-hoc* power analysis showed that we had a power of around 50–90%, for differences in means between groups of around 0.5 to 4 points on cognitive tests (i.e., the range of the effect sizes of the significant findings).

## RESULTS

### Sample characteristics

#### CSF-NfL and Ng concentrations in 70-year-olds with CDR0

Characteristics of participants (*n* = 258) are given in Table 1. Participants had a mean age of 70.6 (SD = 0.3) years, 50% were female and the mean educational length was 13.1 (SD = 3.9) years. Mean and median CSF NfL and Ng levels are given in Table 1. Sociodemographic and clinical characteristics are presented by NfL and Ng status in Table 2, and part of this sample has been published before [2]. In participants with NfL concentrations, no differences were found between those with high versus low levels of NfL. The same applied for high versus low levels of Ng.

**Table 1:** Characteristics of study participants with Clinical Dementia Rating 0 (*n* = 258)

Characteristics
Women, *n* (%)	129 (50)
Age, mean (SD), y	70.6 (0.3)
MMSE score, mean (SD)	29.3 (0.9)
Education, mean (SD), y	13.1 (3.9)
Stroke, *n* (%)	8 (3.1)
Any depression *n* (%)	21 (8.1)
CSF-neurofilament light mean (pg/ml) (SD)	915 (992)
CSF-neurofilament light median (pg/ml)	724
	Min: 276
	Max: 12312
CSF-neurogranin mean (pg/ml) (SD)	206.9 (73.3)
CSF-neurogranin median (pg/ml)	195.7
	Min: 71.7
	Max: 513

A part of this sample has been published before [2].

**Table 2:** Characteristics of the study participants with Clinical Dementia Rating 0 by CSF Neurofilament Light and CSF Neurogranin status

Neurofilament light				Neurogranin		
Characteristics	Below	Above	*p*	Below	Above	*p*
	median	median		median	Median	
	(N = 126)	(N = 130)		(N = 131)	(N = 127)	
Women, *n* (%)	70 (55.6)	58 (44.6)	0.104	63 (48.1)	66 (52.0)	0.618
Age, mean (SD), y	70.5 (0.3)	70.6 (0.2)	0.090	70.5 (0.2)	70.6 (0.3)	0.321
MMSE score, mean (SD)	29.3 (0.9)	29.2 (1.0)	0.400	29.3 (0.9)	29.2 (1.0)	0.566
Education, mean (SD), y	13.2 (4.2)	13.0 (3.6)	0.750	13.2 (3.9)	13.0 (3.9)	0.674
Stroke, *n* (%)	4 (3.2)	3 (2.3)	0.720	4 (3.1)	4 (3.2)	1.000
Any depression *n* (%)	14 (11.1)	7 (5.4)	0.113	8 (6.1)	13 (10.2)	0.260

CSF neurofilament light and CSF neurogranin are compared based on the median, the variables were log-transformed prior to T-test.

### Cognitive performance in participants with CDR0 stratified by high versus low NfL and Ng concentrations

We then compared cognitive test performance between individuals with high versus low CSF NfL levels. Those with higher NfL levels performed worse on tests of memory (Immediate recall, 8.0 versus 8.5 *p* = 0.013, Cohen’s D = 0.31) and language (FAS, 39.9 versus 44.1 *p* = 0.016, Cohen’s D = 0.31) than those with lower NfL levels. No other differences in cognitive test performance were observed in participants with high CSF NfL compared to low CSF NfL (Table 3).

**Table 3:** Cognitive performance in participants with Clinical Dementia Rating 0, stratified by high versus low neurofilament light (NfL) and neurogranin (Ng) concentrations

Neurofilament light						Neurogranin				
	NfL	Mean	NfL	Mean	P1	Ng	Mean	Ng	Mean	P2
	below	score	above	score		below	score	above	score	
	median	(SD)	median	(SD)		median	(SD)	median	(SD)	
**MMSE**	128	29.3 (0.9)	127	29.2 (1.0)	0.364	129	29.3 (0.9)	128	29.2 (1.0)	0.706
**Memory**										
Immediate recall	127	**8.5 (1.5)**	128	**8.0 (1.7)**	**0.013**	129	8.3 (1.5)	128	8.1 (1.7)	0.242
Delayed recall	127	7.9 (1.8)	128	7.5 (1.7)	0.114	129	7.9 (1.7)	128	7.5 (1.7)	0.126
Word memory	124	5.9 (1.7)	127	5.4 (1.8)	0.055	127	5.6 (1.9)	126	5.6 (1.7)	0.961
Supra Span (BUSII)	122	7.8 (1.5)	120	7.6 (1.4)	0.220	122	**7.9 (1.5)**	122	**7.5 (1.4)**	**0.035**
Thurstone’s picture memory test	117	22.8 (3.6)	119	22.8 (4.2)	0.953	118	22.7 (3.9)	119	22.9 (3.8)	0.613
**Language**										
Word fluency	127	25.3 (6.7)	128	24.9 (6.3)	0.664	129	25.4 (6.4)	128	24.8 (6.6)	0.454
FAS	120	**44.1 (14.4)**	123	**39.9 (12.9)**	**0.016**	123	41.9 (13.2)	122	42.0 (14.4)	0.974
**Executive function**										
SRB2	124	20.8 (4.2)	126	20.3 (4.0)	0.325	126	20.8 (4.3)	126	20.3 (3.9)	0.357
Digit span backwards	125	4.5 (1.2)	125	4.7 (1.0)	0.335	128	4.6 (1.2)	124	4.6 (1.1)	0.809
**Visuospatial**										
SRB3	121	22.3 (6.5)	125	21.7 (7.1)	0.411	125	22.3 (6.1)	123	21.5 (7.5)	0.364
**Mental speed**										
Psif	125	30.5 (7.6)	126	28.9 (7.7)	0.098	127	29.8 (7.5)	126	29.7 (7.9)	0.875

NfL and Ng were log-transformed prior to T-tests.

We also examined CSF NfL levels in tertiles. Participants with NfL levels in the highest tertile performed worse on Immediate recall (7.9 versus 8.5, *p* = 0.021, Cohen’s D = 0.37) and Delayed recall (7.4 versus 7.9, *p* = 0.041, Cohen’s D = 0.29) compared to those in the lowest tertile. (Supplementary Table 1).

Since the NfL variable had a skewed distribution, we also performed a Mann-Whitney U-test as a sensitivity analysis (Supplementary Table 4), testing differences in cognitive test scores between individuals with NfL above and below the median. The analyses showed differences between the groups in the Immediate recall (*p* = 0.025) and FAS (*p* = 0.021) tests. The rank-mean was higher in those with NfL below the median for both tests.

Linear regressions with age, education, sex, and APOE *ɛ*4 as covariates were also performed to test for an association between cognition and NfL and Ng, but no associations were found (Supplementary Table 3).

Participants with CSF-Ng above the median performed worse in one memory test compared to those below the median (Supra span, 7.5 versus 7.9 *p* = 0.035, Cohen’s D = 0.28). There were no differences in any other cognitive domains (Table 3).

We then examined CSF Ng in tertiles and detected no differences in cognitive test performance in participants with the highest Ng tertile compared to the lowest Ng tertile. (Supplementary Table 2). We found no association between cognitive test scores and Ng. (Supplementary Table 3), nor did we find a difference between high versus low Ng for any of the cognitive test results in a Mann-Whitney U-test (Supplementary Table 4).

### Cognitive performance in 70-year-olds with CDR0 and biomarker evidence of AD pathology stratified by high versus low NfL and Ng concentrations

To compare participants with and without preclinical AD pathology, we stratified the group by high and low levels of NfL and Ng, and divided these groups based on Aβ_42_ and tau concentrations (the p-tau and t-tau groups merged, since the p-tau group was very small). The group with pathologic tau concentrations may contain participants who also have pathologic amyloid concentrations and vice versa, as this division was necessary to avoid small groups. In the group with high NfL, participants with pathologic tau performed worse on Delayed recall (7.1 versus 8.1, *p* = 0.003, Cohen’s D = 0.64) than the group without normal tau, and the amyloid pathology group also performed worse than the group without amyloid pathology on Delayed recall (7.3 versus 8.1, *p* = 0.044, Cohen’s D = 0.45). There were no differences in any other cognitive tests (Table 4).

**Table 4:** Cognitive performance in 70-year-olds with Clinical Dementia Rating 0 and pathology, stratified by high and low NfL level

NfL above median							NfL below median					
	Amyloid	No amyloid	p1	Pathologic	Normal	p2	Amyloid	No amyloid	p1	Pathologic	Normal	p2
	pathology	pathology		tau	tau		pathology	pathology		tau	tau	
	(N = 36)	(N = 48)		(N = 61)	(N = 48)		(N = 22)	(N = 87)		(N = 24)	(N = 87)	
	Mean (SD)	Mean (SD)		Mean (SD)	Mean (SD)		Mean (SD)	Mean (SD)		Mean (SD)	Mean (SD)	
MMSE	29.2 (1.0)	29.2 (0.8)	0.752	29.0 (1.2)	29.2 (0.8)	0.295	29.2 (0.8)	29.3 (0.9)	0.558	29.5 (0.6)	29.3 (0.9)	0.473
Memory												
Immediate recall	8.0 (1.8)	8.2 (1.6)	0.568	7.7 (1.8)	8.2 (1.6)	0.131	8.5 (1.0)	8.5 (1.7)	0.851	8.7 (1.1)	8.5 (1.7)	0.393
Delayed recall	**7.3 (2.0)**	**8.1 (1.5)**	**0.044**	**7.1 (1.6)**	**8.1 (1.5)**	**0.003**	8.0 (1.5)	7.8 (1.8)	0.745	8.1 (1.7)	7.8 (1.8)	0.463
Word memory	5.2 (1.9)	5.5 (1.9)	0.540	5.4 (1.8)	5.5 (1.9)	0.863	5.9 (1.7)	5.9 (1.8)	0.972	5.8 (1.2)	5.9 (1.8)	0.894
Supra span (BUSII)	7.4 (1.3)	7.9 (1.3)	0.133	7.4 (1.5)	7.9 (1.3)	0.102	8.3 (1.1)	7.8 (1.5)	0.059	7.6 (1.8)	7.8 (1.5)	0.564
Thurstone’s												
Picture memory test	21.4 (5.6)	23.00 (3.8)	0.171	22.4 (4.2)	23.0 (3.8)	0.510	22.7 (3.9)	23.0 (3.5)	0.695	22.6 (4.0)	23.0 (3.5)	0.675
Language												
Word fluency	25.0 (6.2)	25.4 (6.6)	0.773	24.6 (6.1)	25.4 (6.6)	0.472	26.7 (7.0)	25.1 (6.8)	0.340	23.7 (6.5)	25.1 (6.8)	0.380
FAS	40.3 (11.1)	39.9 (11.5)	0.866	38.8 (13.7)	39.9 (11.5)	0.660	46.7 (15.5)	43.0 (14.2)	0.315	48.5 (14.2)	43.0 (14.2)	0.127
Executive function												
SRB2	20.19 (3.7)	20.3 (4.6)	0.889	20.2 (3.7)	20.3 (4.6)	0.825	21.0 (4.6)	20.7 (4.2)	0.778	21.3 (4.3)	20.7 (4.2)	0.513
Digit span backward	4.7 (1.0)	4.8 (0.9)	0.635	4.6 (1.1)	4.8 (0.9)	0.477	4.7 (1.1)	4.6 (1.3)	0.587	4.5 (1.1)	4.6 (1.3)	0.801
Visuospatial												
SRB3	22.0 (6.9)	22.4 (6.9)	0.776	20.5 (7.4)	22.4 (6.9)	0.177	23.3 (6.9)	22.1 (6.5)	0.488	22.2 (7.2)	22.1 (6.5)	0.959
Mental speed												
Psif	27.8 (9.4)	28.8 (5.8)	0.570	29.3 (8.8)	28.8 (5.8)	0.717	30.7 (6.7)	30.6 (8.2)	0.945	29.6 (6.5)	30.6 (8.2)	0.618

Pathologic tau was defined as the t-tau and p-tau groups combined. No amyloid pathology and no tau pathology were defined as A-T-N-.

We also compared participants with pathologic Aβ_42_ and tau to those with normal concentrations stratified by high/low Ng in the same way. In the high Ng level group, participants with amyloid pathology performed worse on the MMSE (29.0 versus 29.4, *p* = 0.027, Cohen’s D = 0.46) than participants without amyloid pathology. There were no other differences in any other cognitive tests (Table 5).

**Table 5:** Cognitive performance in 70-year-olds with Clinical Dementia Rating 0 and pathology, stratified by high and low Ng level

Ng above median							Ng below median					
	Amyloid	No amyloid	p1	Pathologic	Normal	p2	Amyloid	No amyloid	p1	Pathologic	Normal	p2
	pathology	pathology		tau	tau		pathology	pathology		tau	tau	
	(N = 30)	(N = 41)		(N = 80)	(N = 41)		(N = 29)	(N = 97)		(N = 7)	(N = 97)	
	Mean (SD)	Mean (SD)		Mean (SD)	Mean (SD)		Mean (SD)	Mean (SD)		Mean (SD)	Mean (SD)	
MMSE	**29.0 (1.0)**	**29.4 (0.7)**	**0.027**	29.1 (1.1)	29.4 (0.7)	0.115	29.4 (0.8)	29.2 (1.0)	0.346	29.7 (0.5)	29.2 (1.0)	0.194
Memory												
Immediate recall	8.0 (1.7)	8.4 (1.8)	0.349	8.0 (1.7)	8.4 (1.8)	0.146	8.3 (1.4)	8.4 (1.6)	0.878	8.3 (1.3)	8.4 (1.6)	0.904
Delayed recall	7.4 (1.9)	7.9 (1.7)	0.294	7.4 (1.7)	7.9 (1.7)	0.176	7.7 (1.8)	7.9 (1.7)	0.582	7.7 (1.6)	7.9 (1.7)	0.753
Word memory	5.4 (1.8)	5.9 (1.8)	0.305	5.6 (1.7)	5.9 (1.8)	0.396	5.5 (1.8)	5.7 (1.9)	0.594	5.1 (1.8)	5.7 (1.9)	0.468
Supra span (BUSII)	7.7 (1.3)	7.8 (1.3)	0.797	7.4 (1.5)	7.8 (1.3)	0.237	8.0 (1.4)	7.9 (1.5)	0.718	7.6 (2.0)	7.9 (1.5)	0.642
Thurstone’s Picture memory test	21.5 (5.7)	23.7 (3.0)	0.075	22.6 (4.2)	23.7 (3.0)	0.143	22.3 (4.3)	22.8 (3.8)	0.534	23.1 (4.3)	22.8 (3.8)	0.817
Language												
Word fluency	25.2 (7.2)	25.6 (7.2)	0.803	24.0 (6.2)	25.6 (7.2)	0.202	25.9 (5.9)	25.2 (6.6)	0.606	27.4 (5.0)	25.2 (6.6)	0.380
FAS	42.5 (12.9)	43.0 (14.7)	0.887	41.3 (14.5)	43.0 (14.7)	0.549	42.3 (13.4)	42.0 (13.1)	0.895	40.0 (12.2)	42.0 (13.1)	0.705
Executive function												
SRB2	20.4 (3.9)	20.0 (4.1)	0.692	20.3 (3.9)	20.0 (4.1)	0.715	20.7 (4.1)	20.8 (4.4)	0.886	22.3 (3.5)	20.8 (4.4)	0.379
Digit span backward	4.7 (1.1)	4.7 (1.2)	0.970	4.6 (1.1)	4.7 (1.2)	0.461	4.6 (1.0)	4.6 (1.2)	0.991	4.4 (1.4)	4.6 (1.20)	0.746
Visuospatial												
SRB3	22.3 (7.6)	22.0 (8.0)	0.887	21.3 (7.4)	22.0 (8.0)	0.634	22.8 (6.1)	22.2 (6.1)	0.652	19.3 (7.0)	22.2 (6.1)	0.234
Mental speed												
Psif	28.5 (9.7)	30.2 (7.1)	0.400	29.6 (8.2)	30.2 (7.1)	0.703	29.8 (7.5)	29.8 (7.6)	0.990	27.4 (7.2)	29.8 (7.6)	0.431

Pathologic tau was defined as the t-tau and p-tau groups combined. No amyloid pathology was defined as A-T-N-.

**Fig. 1:**
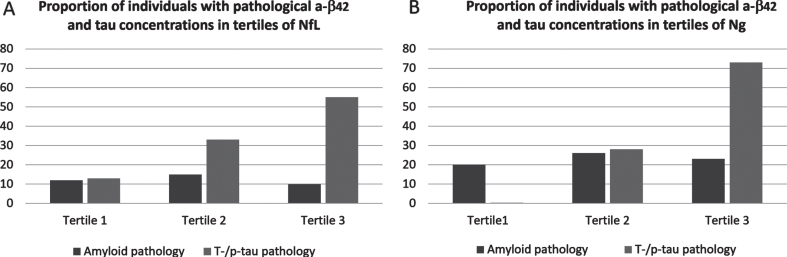
Proportions of participants with pathological concentrations of Aβ_42_ and tau in tertiles of NfL (A) and tertiles of Ng (B).

We investigated the relationship between pathological tau and Aβ_42_ concentrations to NfL and Ng concentrations by dividing all participants into tertiles based on their NfL and Ng concentrations. Participants with higher NfL concentrations more often had pathological t-/p-tau concentrations (13%, 33% and 55% in tertile 1, 2, and 3 respectively) (Fig. 1A). The same pattern was seen in the Ng tertiles where 0%, 28%, and 73% in tertile 1, 2, and 3 had pathological t-/p-tau concentrations (Fig. 1B). This pattern was less clear in relation to Aβ_42_ concentrations where NfL tertile 1, 2, and 3 had 12%, 15%, and 10% of participants with pathological Aβ_42_ concentrations (Fig. 1A). In the Ng tertiles 20%, 26%, and 23% had pathological Aβ_42_ concentrations (Fig. 1B).

### Correction for multiple testing

When performing correction for multiple testing using the Hochberg method, none of the significant p values remained, except the result for delayed recall in Table 4 (*p* = 0.003).

## DISCUSSION

This study examined neurocognitive test performance in cognitively healthy older adults from the general population with and without markers of neurodegeneration (high versus low CSF NfL) and synaptic degeneration (high versus low Ng). Aside from subtle differences in a few cognitive tests, we observed similar test performance in participants with both high and low levels of these biomarkers. However, participants with high NfL performed slightly worse in the memory and the language domain, and those with high Ng performed slightly worse in one memory test, which is in line with our hypothesis, although the effect sizes were small.

The results of our study are in keeping with the results of the cross-sectional population-based study in older adults with normal cognition from the Vanderbilt Memory & Aging Project in Nashville, USA, that found an association between CSF NfL and memory [41], although participants were slightly older than in our study (mean age 72±7 years). Another population-based study from the Mayo Clinic of older adults without dementia investigated associations between CSF and plasma NfL and cognition in five different domains. Although no cross-sectional associations were found between cognition and CSF and plasma NfL, the authors reported longitudinal associations between plasma NfL and worsening cognition, as well as neurodegeneration [42]. As our study is cross-sectional it may be that cognitive decline appears over time in the groups with underlying neurodegeneration as reflected in higher CSF NfL levels, a notion which is supported by the slight decline in two cognitive domains in our study.

Our finding of slightly worse memory in participants with high Ng levels are in line with another cross-sectional study which also found a relationship between Ng and memory in cognitively healthy older adults [43]. In their study, the association between Ng and memory was independent of other AD biomarkers.

Apart from testing in the memory and language domain, we were, however, unable to detect differences between the groups in the majority of the neuropsychological tests, as has been shown before in preclinical AD [44]. Moreover, some findings could be due to multiple testing. The result that participants with NfL levels above median had worse scores in the tests Immediate recall and FAS was also supported by the Mann-Whitney U-test sensitivity analysis. Immediate recall was also significant when dividing NfL into tertiles, where those with the highest NfL performed worse than the reference group (the lowest tertile), indicating that higher levels of NfL indeed reflect more ongoing neurodegeneration. However, in the linear regressions with covariates we could not detect any association between NfL or Ng and any of the cognitive tests, indicating that potential effects of NfL and Ng on test performance are not independent of these covariates.

When we examined participants with underlying preclinical AD pathology in relation to high NfL, we found that having pathologic amyloid and tau concentrations in CSF was related to worse performance in another memory test (Delayed recall). Higher t- or p-tau concentrations is related to ongoing neurodegeneration which is as well reflected in higher NfL levels. We could show that more participants with tau-pathology were in the higher tertiles of NfL whereas more participants with low NfL did not have tau-pathology, indicating that both NfL and tau-pathology relate to the ongoing neurodegeneration. Regarding Ng, we could show that a large portion of participants in the highest tertile of Ng had pathologic tau concentrations while participants in the lower tertiles less often had pathologic tau concentrations, indicating a relationship between neurodegeneration and synaptic pathology. Regarding amyloid pathology, there was a much smaller difference between the highest and lowest tertiles of NfL and Ng. CSF NfL seems to be a marker of unspecific neurodegeneration independent of amyloid pathology, which has been shown before [5].

Since NfL is a marker of neurodegeneration, it seems that a higher level of neurodegeneration is associated with subtly worse cognition in some cognitive tests in otherwise cognitively healthy individuals. High Ng levels were also related to lower performance on a memory test. Synaptic degeneration is an early event in AD. Our results suggest that neuronal damage precedes cognitive decline and that NfL and Ng might be early markers. It is possible that participants with signs of neurodegeneration or synaptic damage may be closer to a conversion to MCI or dementia, although this can only be evaluated in longitudinal data.

Contrary to our hypothesis, there were no differences between individuals with and without preclinical AD pathology, in groups with high NfL level or low NfL level, except that individuals with high NfL levels and preclinical AD (pathologic amyloid and tau concentrations) performed worse in the memory test Delayed recall, and those with Ng and amyloid pathology performed worse in the MMSE. It may be that our participants are so very early in the disease phase (as indicated by the high mean MMSE score) that only subtle differences can be detected.

The results of our study should be interpreted with caution considering the fact that most results did not survive Hochberg correction. However, this method is conservative and may increase the risk of type II errors.

### Strengths and limitations

Among the strengths of this study are the comprehensive examinations and the relatively high response rate for LP. Dementia diagnoses were determined by medical and psychiatric experts, and the examinations were performed by experienced research nurses trained by the Principal Investigator. The inter-rater reliability between nurses and psychiatrists diagnosing dementia was high, as previously reported [45].

As for limitations, participants may have perceived the examinations as wearying which might possibly have an effect during cognitive examinations, but our impression is that this has been a minor issue [23].

In observational studies, there could be a potential for selection bias. We have tried to minimize this bias by the systematic selection of participants and the population-based design, which makes our study more representative of the general population than some other studies that rely on convenience samples or volunteers. The nature of the examinations with a long examination time risks leading to a non-response bias with less healthy individuals remaining at home while healthier persons may be more willing to participate. In an effort to prevent this, we offered home visits. Exclusion from LP due to contraindications might also have biased the sample towards healthier persons. Another limitation is that occurrence of spurious significances due to multiple testing cannot be ruled out. Further, it is possible that the study is underpowered in cases where the cohort was divided into small groups.

The relatively young age and health of the participants could be perceived both as a strength and a limitation— given the sparse studies on NfL and Ng in the general population, this sample could provide novel information about cognitive performance in older adults, but nevertheless differences are challenging to detect at such a relatively young age. The cross- sectional design is also a weakness, and a follow-up will be necessary to determine which participants will proceed to develop cognitive decline and AD in the future. Lastly, these findings cannot be extrapolated to other age groups or other nationalities.

### Conclusions

This study showed that 70-year-olds with markers of neurodegeneration and synaptic pathology had slightly worse performance in some memory tests, although we could not detect differences in other cognitive tests between individuals with high and low NfL or Ng in most of the tests in the battery. Since being cognitively healthy according to CDR was an inclusion criterion, larger differences between participants could not be expected in this study.

## Supplementary Material

Supplementary Material
