# Supplementary material for: Subtle Differences in Cognition in 70-Year-Olds with Elevated Cerebrospinal Fluid Neurofilament Light and Neurogranin: A H70 Cross-Sectional Study
Source: J Alzheimers Dis. 2023 Jan 3;91(1):291–303. doi: 10.3233/JAD-220452 (PMC9881027; doi:10.3233/JAD-220452)
Supplement: Supplementary Material [file jad-91-jad220452-s001.pdf]

Supplementary Material

Subtle Differences in Cognition in 70-Year-Olds with Elevated Cerebrospinal Fluid Neurofilament Light and Neurogranin: A H70 Cross-Sectional Study

Supplementary Table 1. Cognitive performance in 70-year-olds with Clinical Dementia Rating 0 - CSF neurofilament light (NfL) divided into tertials (n = 256)

	**1st T N=84**	**1st T Mean (SD)**	**2nd T N=85**	**2nd T Mean (SD)**	**p**	**3rd T N=87**	**3rd T Mean (SD)**	**p**
MMSE	84	29.3 (0.9)	85	29.2 (1.0)	0.460	86	29.2 (0.9)	0.328
**Memory**								
Immediate recall	84	**8.5 (1.5)**	84	8.3 (1.6)	0.627	87	**7.9 (1.7)**	**0.021**
Delayed recall	84	**7.9 (1.6)**	84	7.8 (1.7)	0.716	87	**7.4 (1.8)**	**0.041**
Word memory	83	5.8 (1.8)	82	5.5 (1.5)	0.298	86	5.6 (2.0)	0.374
Supra Span	82	7.7 (1.6)	80	7.8 (1.3)	0.769	80	7.6 (1.4)	0.650
Thurstone's picture memory test	78	22.9 (3.4)	78	22.4 (4.2)	0.400	80	23.1 (4.1)	0.750
**Language**								
Word fluency	84	25.6 (7.3)	84	25.2 (5.7)	0.682	87	24.5 (6.3)	0.321
FAS	82	43.7 (14.1)	79	42.3 (13.3)	0.525	82	39.9 (13.9)	0.083
**Executive function**								
SRB2	83	20.2 (4.1)	82	20.8 (4.1)	0.378	85	20.5 (4.2)	0.677
Digit span backwards	84	4.5 (1.2)	82	4.6 (1.2)	0.589	84	4.7 (1.0)	0.261
**Visuospatial**								
SRB3	83	22.3 (6.6)	79	21.5 (6.2)	0.476	84	21.9 (7.5)	0.742
**Mental speed**								
Psif	84	29.5 (7.8)	82	30.8 (7.4)	0.277	85	28.8 (7.7)	0.565

T, tertile. The 1st tertile was the reference group.
NfL was log-transformed before divided into tertiles.

Supplementary Table 2. Cognitive performance in 70-year-olds with Clinical Dementia Rating 0 - CSF-Neurogranin (Ng) divided into tertiles (n = 258)

	**1st T N=91**	**1st T Mean (SD)**	**2nd T N=76**	**2nd T Mean (SD)**	**p**	**3rd T N=91**	**3rd T Mean (SD)**	**p**
MMSE	91	29.2 (1.0)	76	29.3 (0.8)	0.471	90	29.2 (1.0)	0.673
**Memory**								
Immediate recall	91	8.2 (1.6)	76	8.0 (1.8)	0.475	90	8.3 (1.5)	0.623
Delayed recall	91	7.8 (1.7)	76	7.5 (1.9)	0.244	90	7.8 (1.6)	0.780
Word memory	90	5.6 (1.8)	75	5.5 (1.8)	0.843	88	5.7 (1.8)	0.701
Supra Span	86	7.8 (1.4)	72	7.7 (1.5)	0.644	86	7.6 (1.5)	0.245
Thurstone's picture memory test	84	22.6 (4.0)	68	22.9 (4.3)	0.662	85	22.8 (3.4)	0.751
**Language**								
Word fluency	91	25.2 (6.6)	76	25.3 (7.1)	0.904	90	24.8 (5.9)	0.660
FAS	87	41.8 (13.2)	73	41.8 (13.2)	0.990	85	42.3 (14.9)	0.824
**Executive function**								
SRB2	89	20.6 (4.1)	75	20.9 (4.2)	0.657	88	20.2 (4.0)	0.557
Digit span backwards	90	4.7 (1.2)	76	4.5 (1.1)	0.224	86	4.6 (1.1)	0.574
**Visuospatial**								
SRB3	88	22.2 (5.7)	75	22.0 (7.7)	0.843	85	21.4 (7.0)	0.402
**Mental speed**								
Psif	89	29.4 (7.6)	76	30.0 (8.0)	0.605	88	29.8 (7.5)	0.694

T, tertile. The 1st tertile was the reference group
Ng was log-transformed before divided into tertiles.

Supplementary Table 3. Linear regression with cognitive tests as dependent variables and Neurofilament light or Neurogranin as independent variables in a population-based sample of 70-year-olds with Clinical Dementia Rating 0

**Model 1:** No covariates				
	**Neurofilament light**		**Neurogranin**	
**Cognitive tests**	**Standardized β**	**p**	**Standardized β**	**p**
MMSE	0.065	0.298	-0.049	0.432
Immediate recall	0.010	0.874	-0.015	0.808
Delayed recall	<0.001	0.994	-0.027	0.664
Word memory	0.030	0.633	0.026	0.686
Supra Span	-0.010	0.883	-0.021	0.748
Thurstone's picture memory test	0.123	0.059	0.023	0.724
Word fluency	0.043	0.491	-0.016	0.796
FAS	0.001	0.986	-0.012	0.852
SRB2	-0.002	0.976	-0.054	0.396
Digit span backwards	0.038	0.551	-0.035	0.582
SRB3	0.031	0.625	-0.087	0.173
Psif	-0.023	0.713	0.021	0.734
**Model 2:** Age, education, sex, and *APOE* ε4 as covariates				
**Cognitive tests**	**Standardized β**	**p**	**Standardized β**	**p**
MMSE	0.066	0.295	-0.026	0.683
Immediate recall	0.009	0.884	-0.024	0.703
Delayed recall	-0.005	0.934	-0.037	0.558
Word memory	0.031	0.631	0.028	0.660
Supra Span	-0.018	0.782	-0.035	0.586
Thurstone's picture memory test	0.123	0.056	0.037	0.571
Word fluency	0.046	0.463	0.012	0.850
FAS	0.001	0.992	0.005	0.940
SRB2	-0.001	0.993	-0.026	0.671
Digit span backwards	0.037	0.564	-0.018	0.774
SRB3	0.037	0.546	-0.062	0.318
Psif	-0.024	0.706	0.054	0.393

Supplementary Table 4. Mann-Whitney U-test with neurofilament light and neurogranin divided by the median in cognitive performance in 70-year-olds with Clinical Dementia Rating 0

	**NfL**	**Ng**
**Cognitive tests**	**Significance**	**Significance**
MMSE	0.574	0.557
Immediate recall	**0.025**	0.392
Delayed recall	0.164	0.055
Word memory	0.154	0.969
Supra Span	0.151	0.110
Thurstone's picture memory test	0.709	0.728
Word fluency	0.767	0.407
FAS	**0.021**	0.847
SRB2	0.592	0.275
Digit span backwards	0.165	0.653
SRB3	0.632	0.380
Psif	0.135	0.874
